# Clinical and Patient Comparison of AI and Expert Digital Smile Design: A Prospective Paired Study

**DOI:** 10.3390/dj14030166

**Published:** 2026-03-12

**Authors:** Thamer Almohareb, Asmaa Abou-Bakr, Fatma E. A. Hassanein, Yousra Ahmed, Mostafa Hamza, Mohamed Aboheikal, Nermeen Nagi

**Affiliations:** 1 Department of Restorative Dental Science, College of Dentistry, King Saud University, Riyadh 11451, Saudi Arabia; talmohareb@ksu.edu.sa; 2 Department of Oral Medicine and Periodontology, Faculty of Dentistry, Galala University, Suez 4356111, Egypt; asmaa.abdalraouf@gu.edu.eg; 3 Department of Oral Medicine, Periodontology, and Oral Diagnosis, Faculty of Dentistry, King Salman International University, South Sinai 46618, Egypt; 4 Department of Prosthodontics, Faculty of Dentistry, King Salman International University, South Sinai 46618, Egypt; yousra.elsayed@ksiu.edu.eg; 5 Department of Prosthodontics, Faculty of Dentistry, Galala University, Suez 4356111, Egypt; mostafa.hamza@gu.edu.eg (M.H.); mohamed.abouheikal@gu.edu.eg (M.A.); nermeen.saad@gu.edu.eg (N.N.); 6 Faculty of Dentistry, Fayoum University, 63514 Fayoum, Egypt

**Keywords:** artificial intelligence, SmileFy, digital smile design, dental esthetics, patient preference, digital dentistry

## Abstract

**Background**: Artificial intelligence (AI) systems are increasingly being used in digital smile design and esthetic treatment planning; however, evidence comparing the esthetic performance of AI-generated designs with expert clinician-generated designs remains limited. Objective evaluation using standardized esthetic indices is necessary to determine whether AI-generated outcomes achieve comparable clinical quality. **Methods**: This prospective paired comparative study included 33 patients. For each case, two smile designs were created: one generated using a fully automated AI system (SmileFy) and one designed manually by an experienced clinician using Exocad software(version 3.2 Elefsina; exocad GmbH, Darmstadt, Germany). Twenty blinded prosthodontists evaluated all designs using the Dental Esthetic Screening Index (DESI) and a visual analog scale (VAS). Patients provided esthetic VAS ratings and forced-choice preferences. Objective geometric measurements and total design time were recorded. Paired statistical analyses were performed with a significance level of *p* < 0.05. **Results**: AI-generated designs demonstrated significantly lower total DESI scores than expert-generated designs (14.79 ± 1.63 vs. 18.73 ± 1.82; *p* < 0.001). Both expert and patient VAS ratings were significantly higher for AI designs (*p* < 0.001). Patients preferred AI-generated designs in 69.7% of cases compared with 30.3% for expert designs (*p* < 0.001). AI workflows were significantly faster, with a mean design time of 30.82 ± 5.14 min versus 63.48 ± 14.12 min for expert workflows, corresponding to a 51.46% reduction in planning time (*p* < 0.001). **Conclusions**: Fully automated AI-generated smile designs demonstrated favorable esthetic performance, higher patient acceptance, and substantial improvements in workflow efficiency compared with expert-driven digital designs, supporting their potential role as adjunctive tools in esthetic treatment planning.

## 1. Introduction

The integration of digital technology has transformed diagnostic and treatment planning protocols in esthetic dentistry, and DSD has established itself as a cornerstone in predictable and patient-centered outcomes [1]. This digital paradigm makes use of photographic and scan-based information to evaluate dentofacial relationships, simulate outcomes, and enhance communication beyond the limitations of subjective, analog techniques [2].

The traditional digital smile design (DSD) workflow, when performed by expert clinicians using advanced software such as Exocad DentalCAD, is a very detailed process requiring substantial time, technical expertise, and deep knowledge of esthetic principles [3].

Artificial intelligence (AI) and machine learning (ML) mark the beginning of a new era in the automation of dentistry, from diagnostics to treatment planning [4,5,6,7]. In smile design, totally automated AI systems promise to create personalized smile proposals within seconds by analyzing facial and dental landmarks, thereby shifting the paradigm from manually, expert-dependent creation to algorithm-based generation [8]. This technological development is bringing a crucial clinical question to the forefront: Can the esthetic quality of AI-generated smile designs match that of those meticulously created by an experienced clinician?

Though recent systematic reviews have outlined the ever-growing application of AI in different dental disciplines, a scientific and direct comparison between fully automated AI smile design and an expert-driven conventional digital workflow is strikingly missing from the existing literature [9,10,11]. Several studies have focused on the diagnostic applications of AI in caries detection and periodontal disease assessment, whereas its role in creative design and esthetic treatment planning remains comparatively less explored [12,13,14,15].

Despite the fast evolution of digital workflows, a critical gap remains in how fully automated AI-generated smile designs compare to the expert-driven conventional digital workflow before any restorative intervention is initiated. Smile design is the foundational step that guides diagnostic communication and treatment planning and, ultimately, fabrication of definitive restorations; therefore, any error or bias produced at this level may be propagated throughout the rehabilitative process. The evaluation of AI-generated designs at the virtual level has clinical relevance in deciding whether such emerging technologies can reliably produce esthetic proposals that meet professional standards and patient expectations before irreversible tooth preparation or restorative fabrication is undertaken. It is essential to establish this base evidence before integrating automated AI systems into clinical decision making or allowing them to influence restorative outcomes.

This prospective study was therefore designed to conduct a direct comparative analysis of smile designs generated by an expert clinician using Exocad software versus fully automated AI system designs (SmileFy). The comparison was made from multiple standpoints: objective esthetic parameters, subjective ratings by both patients and prosthodontists, design preference, and time needed for completion. The null hypothesis considered that no significant differences would exist between the two methodologies concerning esthetic quality, preference, or time needed for completion.

## 2. Materials and Methods

### 2.1. Study Design and Ethical Approval

This prospective comparative experimental study evaluated esthetic outcomes of AI-generated versus expert-generated smile designs. The study was conducted in a digital dentistry laboratory using standardized photographic protocols and calibrated digital design software. The study followed the STROBE guidelines for observational research. Ethical approval was obtained from the Faculty of Dentistry, Fayoum University (Approval No. R 753). All participants signed written informed consent permitting the use of their clinical photographs, scans, and mock-ups for research purposes.

### 2.2. Participants and Case Selection

Patients were recruited consecutively from the esthetic dentistry outpatient clinic until the required sample size of patients was achieved. Individuals presenting with anterior spacing, disharmony in tooth proportions, or dissatisfaction with their smile esthetics were screened for eligibility.

### 2.3. Sample Size Calculation

The sample size was calculated for a paired comparison of DESI scores at the case level. Preliminary estimates were derived from a pilot analysis of five cases conducted prior to the main study. The pilot data suggested an expected mean paired difference of 2 points with a standard deviation of 4. Assuming a two-sided α of 0.05 and 80% statistical power, the required sample size was estimated to be approximately 32 cases using the standard formula for paired mean comparisons. Accordingly, a target sample size of 30–35 patients was selected to ensure adequate power. The pilot cases were not included in the final analysis. The number of expert raters was set at 20 to enhance reliability and robustness of scoring. Previous DESI validation studies have demonstrated excellent reliability with as few as five raters; therefore, inclusion of 20 raters was considered methodologically sufficient.

### 2.4. Inclusion and Exclusion Criteria

Eligible participants were adults aged 18 to 50 years with intact maxillary anterior teeth, good oral hygiene, and stable periodontal health. They were required to have an esthetic concern involving the anterior region and to be capable of completing all digital and clinical mock-up procedures. Patients were excluded if they had active periodontal disease, caries, or large restorations affecting the esthetic zone, recent orthodontic or prosthodontic treatment, craniofacial anomalies, significant facial asymmetry, parafunctional habits, or contraindications to adhesive mock-up bonding. Patients unwilling to permit clinical photography or the use of their digital data were also excluded.

### 2.5. Digital Smile Design Workflows

**Figure 1:**
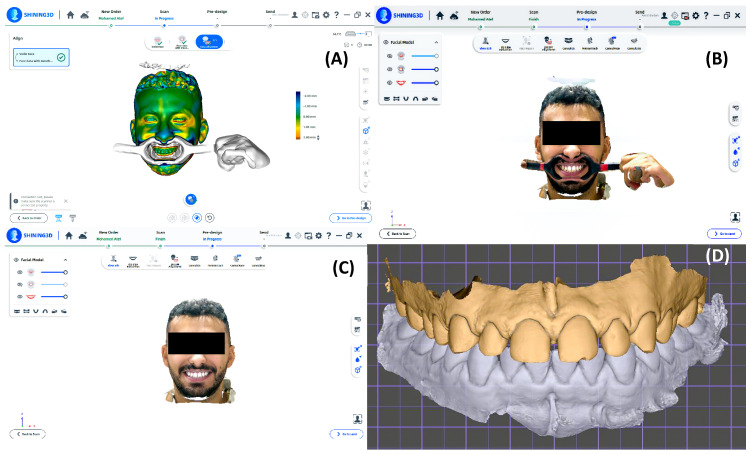
Digital data acquisition and integration workflow for smile design planning. (**A**) Full-face three-dimensional facial scan acquired in a smiling position to capture facial morphology and dentofacial landmarks. (**B**) Facial scan obtained with cheek retractors to visualize maxillary dentition and gingival display for alignment purposes. (**C**) Oriented facial scan in natural smiling position following preprocessing and alignment. (**D**) Superimposition of the maxillary intraoral scan onto the facial scan to integrate facial and dental data into a unified digital model for smile design generation. Images represent digital input data and were minimally processed by cropping and uniform global adjustment without alteration of anatomical features.

For each patient included, comprehensive digital records were obtained. Extraoral photographs were captured in standardized frontal, three-quarter, profile, and smile views using a DSLR camera (Nikon D7000, Nikon Corporation, Tokyo, Japan) with120 mm lens, while intraoral photographs were obtained using a Nikon 5100 with a macro 105 mm lens. Maxillary and mandibular scans were acquired digitally using an Omnicam intraoral scanner (Dentsply Sirona, Bensheim, Germany), and a full-face 3D scan was recorded using Metismile (SHINING 3D, Hangzhou, China). All files were exported as JPEG, STL, and OBJ/OPG formats (Figure 1).

Digital smile design relies on stable facial and dental reference landmarks—such as the facial midline, interpupillary line, incisal edges, and gingival margins—that are identifiable in standardized frontal photographs and serve as the foundation for esthetic calibration [16,17].

### 2.6. Digital Data Acquisition

All photographic and digital records were obtained under standardized conditions, including identical camera systems, focal length, lighting environment, background, and patient positioning. No esthetic filters, smoothing algorithms, or selective enhancements were applied to either AI- or expert-generated designs. Images were exported at identical resolution and presented using uniform display settings. Designs were anonymized, randomly labeled (Design A/B), stripped of metadata, and presented in randomized order to both experts and patients, who were blinded to design origin.

### 2.7. Digital Data Preprocessing and Registration Standardization

Prior to design generation, all facial and intraoral scan files underwent standardized preprocessing within the native scanning software environment, including uniform trimming of non-essential peripheral structures, orientation to natural head position using facial reference planes, scale calibration verification, and landmark-based alignment of intraoral scans to facial scans. No morphologic modification, smoothing, or dimensional scaling was performed. All files were exported using identical resolution and mesh-density parameters before import into both AI- and expert-based workflows to ensure consistent registration and prevent systematic bias.

### 2.8. Expert-Generated Digital Smile Design (Design A)

**Figure 2:**
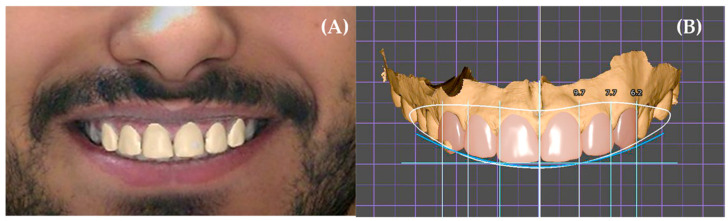
Expert-generated digital smile design workflow (Design A). (**A**) Facial reference image used by the expert clinician to establish midline, smile line, and facial proportions during digital smile design planning. (**B**) Expert-generated digital tooth setup illustrating reference lines and linear measurements applied to guide tooth positioning, proportions, and smile arc development. The colored reference lines represent standard digital smile design guides used for facial midline alignment, interpupillary line orientation, tooth angulation, and incisal edge positioning. Images represent expert-driven design planning stages and were minimally processed by cropping and uniform global adjustment without alteration of anatomical or esthetic features.

Expert-generated designs were created in Exocad DentalCAD following a structured dentofacial esthetic workflow. The protocol included alignment of facial references, selection of tooth libraries, adjustment of tooth morphology, gingival architecture refinement, midline correction, smile arc harmonization, and buccal corridor shaping. All software tools were fully available to the designer to achieve an optimal esthetic outcome. Digital smile design was performed manually by an experienced clinician using facial references, proportional guidelines, and iterative refinement of tooth morphology and position, as shown in Figure 2.

### 2.9. AI-Based Smile Design Generation (Design B)

**Figure 3:**
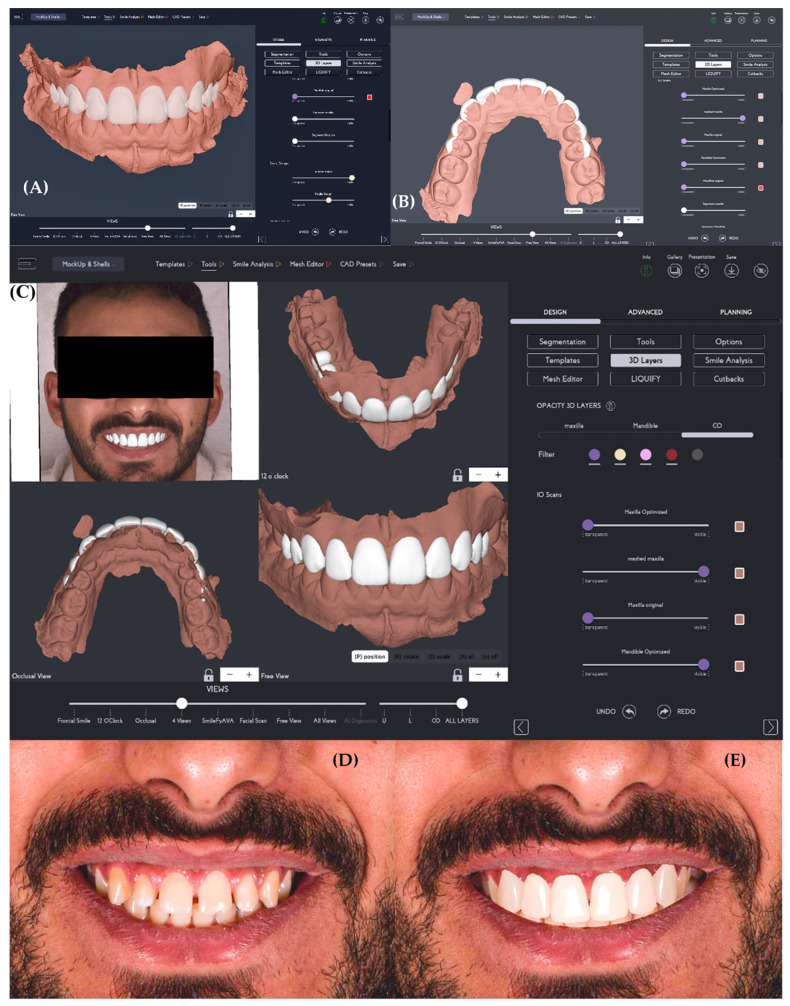
AI-based digital smile design generation workflow (Design B). (**A**) Automated digital tooth setup generated by the AI software based on deep learning-driven analysis of facial and dental landmarks, illustrating the initial esthetic proposal without operator input. (**B**) Occlusal view of the AI-generated maxillary tooth arrangement showing arch form and tooth positioning produced automatically by the system. (**C**) Multi-view visualization of the AI-generated digital smile design allowing three-dimensional assessment of tooth proportions, alignment, and smile arc. (**D**) Pre-design frontal smiling facial photograph used as input for AI analysis. (**E**) Final AI-generated smile design preview superimposed on the facial photograph, representing the fully automated esthetic output. Images represent fully automated AI-generated design stages and were minimally processed by cropping and uniform global adjustment without alteration of anatomical or esthetic features.

AI-generated designs were produced using SmileFy software (version 3.0; SmileFy Inc., Miami, FL, USA). SmileFy software was selected due to its standardized automated design protocol, allowing consistent generation of AI-based esthetic outputs for comparison purposes. The system applied deep learning-based landmark detection to analyze face shape, smile line, gingival display, and tooth proportions. The AI then generated a complete smile proposal without manual intervention. Only the automatic esthetic output was analyzed to preserve methodological consistency (Figure 3).

This particular AI software was selected because it represents one of the few clinically established platforms that are capable of fully automated smile-design proposals right from standardized 2D photographs. Unlike semi-automated systems, this software does not use any manual landmark placements or operator-dependent adjustments; it provides a complete autonomous design output. It therefore allowed for a real comparison between an operator-independent AI workflow and an expert-guided Exocad workflow. Its widespread clinical adoption and standardized output format further secured that the comparison would be reproducible and clinically relevant.

### 2.10. Blinding and Image Presentation Standardization

All photographic and digital records were acquired under standardized conditions, including identical camera systems, fixed focal length, controlled clinical lighting, neutral background, and standardized patient positioning in natural head posture. Smile instructions were uniform for all participants. No smoothing filters, texture enhancement, or selective esthetic post-processing were applied to either AI-generated or expert-generated designs. Images were exported at identical resolution and presented using uniform display settings.

For evaluation, all designs were anonymized and randomly labeled as “Design A” or “Design B.” File names and metadata were removed to eliminate origin disclosure. The presentation order was randomized for each case. Expert evaluators received standardized, anonymized image sets and completed independent scoring forms. Patients viewed both designs under identical viewing conditions, without disclosure of design origin and without clinician guidance, prior to completing VAS ratings and forced-choice preference selection.

### 2.11. Outcome Measures

A panel of 20 prosthodontists served as blind evaluators. Each rater received standardized and anonymized design images for all cases, with the expert and AI designs labeled randomly as “Design A” and “Design B.”

#### 2.11.1. DESI Esthetic Scoring (Primary Outcome)

The Dental Esthetic Screening Index (DESI) [13] was applied to all 66 designs (33 AI and 33 expert). DESI evaluates 12 esthetic components, divided into

Extraoral (5 items):

Facial–dental midline alignment, angulation of upper central incisors, canine line parallelism to the interpupillary line, upper incisor display upon smiling, and lower-lip curvature parallelism.

Intraoral (7 items):

Gingival contour harmony, papilla filling, arch continuity, tooth angulation, proximal contact position, color harmony, and width–height ratio of maxillary central incisors.

Each item was scored 1–5, resulting in a total score range of 12–60. Twenty board-certified prosthodontists independently scored the designs after completing a structured calibration session. Inter-rater reliability (ICC2,1 and ICC2,k) was calculated according to STROBE recommendations for measurement consistency.

#### 2.11.2. Subjective Esthetic Assessment (Expert and Patient VAS Ratings)

A total of 20 blinded experts and 33 patients blindly rated each design using a 0–100 Visual Analog Scale (VAS) [14].

Experts: The same 20 prosthodontists provided independent ratings.

Patients: The original participants scored esthetic appeal and selected their preferred design in a forced-choice task.

VAS was treated as a continuous outcome consistent with STROBE’s recommendation for transparent definition of primary and secondary variables.

#### 2.11.3. Objective Esthetic Measurements

**Figure 4:**
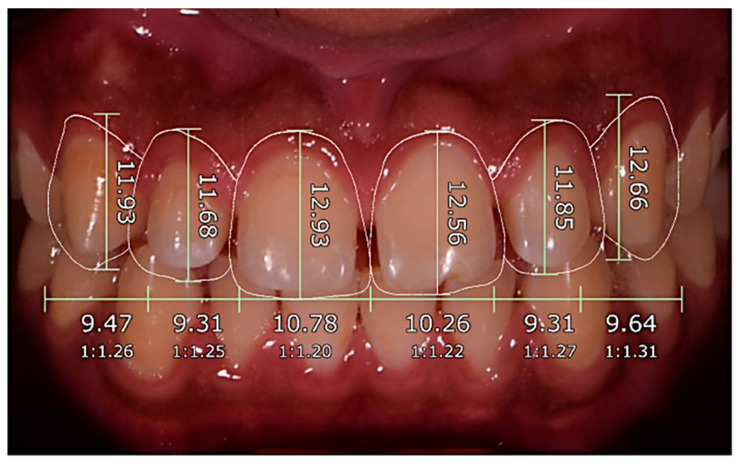
Objective geometric parameters used for smile design assessment. Representative illustration of standardized digital measurements performed on facial and intraoral images for objective esthetic analysis. Measurements included central incisor width and height, width–height ratio, facial–dental midline deviation, gingival zenith symmetry, and buccal corridor percentage, obtained using digital reference lines and calibrated software tools. All measurements were performed by a single calibrated examiner blinded to the design origin (AI-generated or expert-generated) to minimize measurement bias.

Objective geometric parameters were extracted from each design using standardized digital tools, including (Figure 4)

1. Central incisor width (mm);
2. Central incisor height (mm);
3. Width–height ratio (%);
4. Midline deviation (mm);
5. Gingival zenith symmetry (mm difference);
6. Buccal corridor percentage (%).

All measurements were performed by a calibrated examiner blinded to design origin to reduce measurement bias, fulfilling STROBE requirements for blinding where applicable.

#### 2.11.4. Forced-Choice Preference Assessment

Experts and patients completed forced-choice evaluations for each pair (AI vs. expert), selecting the more esthetic design. Preferences were recorded categorically and analyzed using chi-square or McNemar tests as appropriate.

#### 2.11.5. Time Efficiency Assessment

For each patient, the total time required to complete the Exocad design was recorded from the moment of data import until final approval of the wax-up. AI design time was recorded from the moment of file upload until automated generation of the final SmileFy output.

### 2.12. Statistical Analysis

All statistical analyses were performed using R software version 4.X (R Foundation for Statistical Computing, Vienna, Austria) with the tidyverse, psych, irr, ggplot2, and blandr packages, and IBM SPSS Statistics version 23 (IBM Corp., Armonk, NY, USA). Continuous outcomes, including DESI scores, VAS ratings, objective esthetic measurements, and design-time values, were assessed for normality and analyzed using paired *t*-tests. Effect sizes were quantified using Cohen’s *d*. Categorical preference data (AI vs. expert) were analyzed using chi-square or McNemar tests, as appropriate. Inter-rater reliability for DESI and expert VAS scores was evaluated using intraclass correlation coefficients (ICC [2,1] and ICC [2,k]) based on a two-way random-effects model with absolute agreement. Associations among DESI, VAS, objective metrics, and design time were examined using Pearson correlation analysis and visualized with a correlation heatmap generated in R. Agreement between AI- and expert-generated designs for DESI, VAS, and buccal corridor width was assessed using Bland–Altman analysis performed in R. Statistical significance was set at α = 0.05. All analyses were conducted on complete datasets with no missing values.

## 3. Results

### 3.1. Participant Characteristics

**Figure 5:**
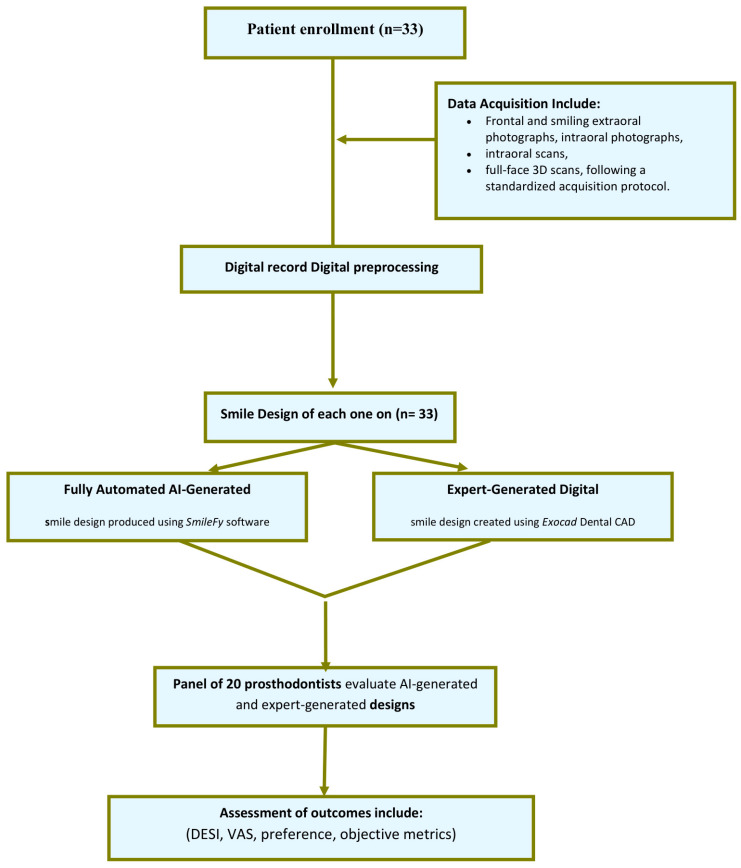
Study workflow diagram.

Thirty-three patients were enrolled in this study. The mean age of the cohort was 29.5 ± 4.3 years, and the sample included 18 males (54.5%) and 15 females (45.5%). The primary esthetic concerns prompting consultation were anterior spacing in 13 patients (39.4%) and general dissatisfaction with smile appearance in 20 patients (60.6%). Baseline esthetic measurements demonstrated a mean midline deviation of 0.58 ± 0.79 mm and a mean gingival display of 1.61 ± 0.83 mm during smiling. All participants completed the full assessment protocol, and no missing demographic or baseline esthetic data were recorded. The overall methodological workflow is summarized in Figure 5.

### 3.2. Expert Rater Characteristics

Twenty board-certified prosthodontists served as expert evaluators in this study, forming a highly experienced assessment panel. The raters had extensive clinical backgrounds in esthetic and restorative dentistry, with an average of 10.8 ± 3.1 years of clinical experience. All evaluators completed a structured calibration session prior to formal data collection, using standardized reference cases and consensus-based scoring anchors.

Inter-rater reliability was assessed using two-way random-effects intraclass correlation coefficients. Calibration showed excellent reliability, with ICC(2,1) = 0.89 for DESI intraoral items, ICC(2,1) = 0.86 for DESI extraoral items, and ICC(2,1) = 0.91 for the overall DESI composite score. Corresponding average-rater reliability values were even higher, with ICC(2,k) = 0.96, 0.94, and 0.97, respectively, confirming strong consistency across the 20-member panel.

For VAS esthetic ratings, reliability was similarly robust. Single-rater agreement reached ICC(2,1) = 0.82, indicating good reliability, while the average-rater ICC(2,k) = 0.95 reflected excellent consistency when scores were aggregated across evaluators. All experts completed the full scoring protocol with no missing data, ensuring a complete and methodologically reliable expert dataset for all cases.

### 3.3. DESI Scores

**Figure 6:**
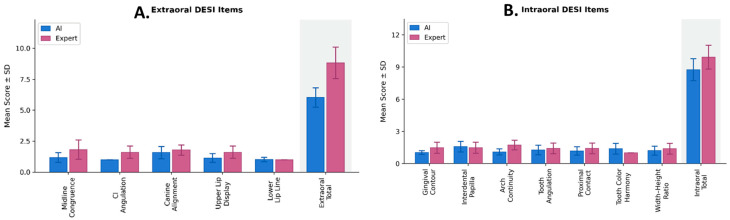
Comparison of DESI item scores between AI-generated and expert designs. Panel (**A**) shows extraoral items and panel (**B**) shows intraoral items. Bars represent mean ± SD. Domain totals reflect the sum of corresponding item scores. Higher scores indicate superior esthetic performance. AI = artificial intelligence.

AI-generated smile designs demonstrated significantly lower DESI scores than expert-generated designs across several esthetic domains (Table 1; Figure 6). AI designs showed superior performance in midline congruence, central incisor angulation, upper-lip tooth display, gingival contour, and arch continuity, with medium to large effect sizes. No significant differences were detected in tooth angulation, interdental papilla, proximal contacts, or width–height ratio. Expert designs performed better only in tooth-color harmony. Overall, AI designs achieved significantly lower extraoral, intraoral, and total DESI scores, indicating more favorable esthetic outcomes compared with expert-generated designs.

### 3.4. Overall VAS Esthetic Ratings

Patients and expert evaluators both assigned high esthetic ratings across all designs; however, patients consistently rated the esthetics higher than clinicians did. The mean VAS score provided by patients was 97.59 ± 1.21, compared with 96.61 ± 0.94 for expert evaluators, producing a statistically significant difference of +0.98 (95% CI, 0.55–1.42; *p* < 0.001, Cohen’s d = 0.56). This reflects a moderate effect size and indicates that patients perceived the esthetic outcomes more positively than expert raters did.

When comparing the two design types, AI-generated designs received higher VAS esthetic ratings than expert-generated designs. The mean VAS score for AI designs was 97.0 ± 0.66, significantly higher than the 96.21 ± 1.02 assigned to expert designs (mean difference = +0.79; 95% CI, 0.51–1.06; *p* < 0.001; Cohen’s d = 1.01). This represents a large effect size, demonstrating a robust preference toward AI-generated smile outcomes based on perceived esthetic quality. Although statistically significant, the absolute magnitude of VAS differences was small and should be interpreted in light of the ceiling effect observed in digitally rendered simulations.

Correlation analysis revealed a significant negative association between patient and expert VAS scores (r = −0.33; *p* = 0.006), indicating differing esthetic perceptions between the two groups. Collectively, these findings confirm that both patients and experts rated AI-generated designs highly, with patients consistently demonstrating the most favorable esthetic evaluations (Table 2).

### 3.5. Correlation Analysis

**Figure 7:**
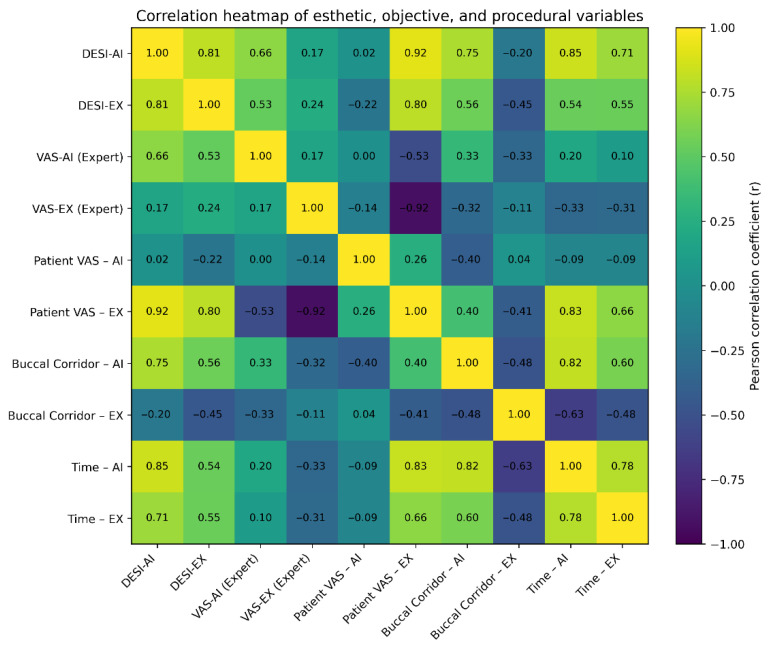
Correlation heatmap illustrating relationships among objective esthetic measures (DESI scores, buccal corridor width, and design time) and subjective ratings (expert and patient VAS) for AI-generated and expert-generated smile designs. Color intensity and numeric annotations represent Pearson correlation coefficients (r).

Correlation analysis revealed distinct patterns linking objective, subjective, and procedural esthetic measures across AI-generated and expert-generated designs (Table 3; Figure 7). DESI scores for AI designs showed strong positive correlations with buccal corridor width (r = 0.75) and design time (r = 0.85), indicating that higher-scoring AI designs tended to include narrower buccal corridors and required longer generation times. DESI-AI demonstrated a moderate correlation with expert VAS ratings (r = 0.66), but little association with patient VAS scores (r = 0.02), confirming that expert-derived esthetic criteria do not directly translate to patient-perceived esthetics.

DESI-EX similarly correlated with patient VAS for expert designs (r = 0.80), yet showed weak relationships with objective parameters. Patient and expert esthetic ratings diverged substantially: patient VAS for expert designs was strongly negatively correlated with expert VAS (r = −0.92), indicating marked differences in esthetic perception between patients and clinicians. Design time correlated strongly with both DESI scores and buccal corridor measurements for AI and expert workflows, suggesting that esthetic complexity—particularly related to arch form and corridor width—increases design time regardless of method. Collectively, these findings support the conclusion that DESI reflects expert-oriented esthetic criteria, whereas subjective VAS ratings capture patient-centered perception, each contributing unique insights into digital smile design evaluation. 

### 3.6. Bland–Altman Agreement Analysis

**Figure 8:**
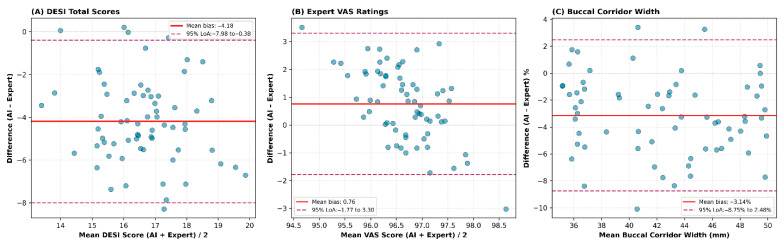
Bland–Altman plots illustrating agreement between AI-generated and expert-generated designs for (**A**) DESI total scores, (**B**) expert visual analog scale (VAS) ratings, and (**C**) buccal corridor width measurements. Solid lines represent mean bias, and dashed lines indicate 95% limits of agreement. No proportional bias was observed across the measurement ranges.

Bland–Altman plots were generated to assess agreement between AI-generated and expert-generated designs across DESI scores, VAS ratings, and buccal corridor measurements, as shown in Figure 8. For DESI total scores, the mean bias was −3.33, with limits of agreement ranging from −7.76 to +1.09, indicating that AI tended to yield slightly lower DESI scores than experts, though remaining within clinically acceptable bounds. Expert VAS ratings demonstrated a small positive bias of +0.79, with narrow limits of agreement (−0.74 to +2.32), reflecting consistent evaluator scoring for AI and expert designs. Buccal corridor width showed the largest systematic difference, with AI designs averaging –3.64% narrower than expert designs and wider limits of agreement (−10.24 to +2.97), consistent with the objective measurement findings. No proportional bias was observed in any comparison, indicating stable agreement across the full measurement range.

### 3.7. Forced-Choice Preference

Patients demonstrated a stronger preference for AI-generated smile designs compared with expert evaluators (Table 4). While experts selected the AI design in 51.5% of cases, patients preferred the AI design in 69.7% of cases. This difference in preference distribution was statistically significant (χ^2^ = 20.44, *p* < 0.001). Agreement between patient and expert choices was modest at 54.5%, and Cohen’s κ indicated no agreement beyond chance (κ = 0.00). McNemar’s test was not interpretable, due to the absence of discordant preference pairs. These findings highlight a clear divergence in esthetic perception, with patients consistently favoring AI-generated designs more strongly than expert evaluators.

Objective Esthetic Measurements

**Figure 9:**
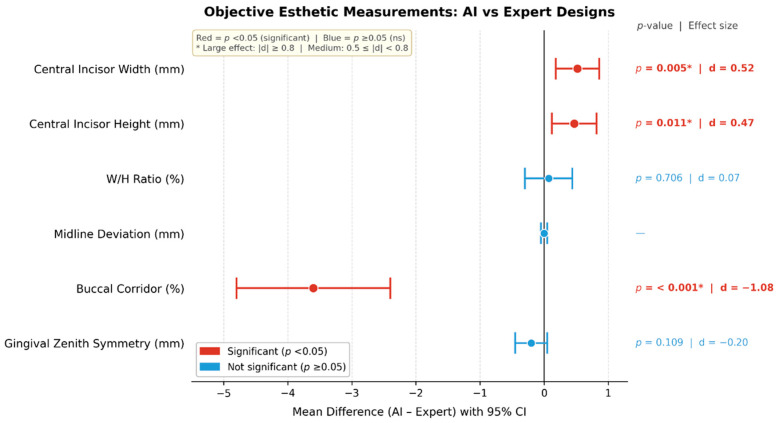
Forest plot illustrating mean differences (AI − Expert) and 95% confidence intervals for objective esthetic measurements comparing AI-generated and expert-generated smile designs. Outcomes are presented in their native units (mm or %), and the x-axis represents raw mean differences that should be interpreted within each parameter. Positive values indicate larger measurements in AI-generated designs, whereas negative values indicate larger measurements in expert-generated designs. Mid-line deviation showed no variability across paired designs. Red markers indicate statistically sig-nificant differences (*p* < 0.05).

Objective esthetic measurements revealed several significant differences between AI-generated and expert-generated smile designs (Table 5; Figure 9). AI designs produced slightly larger central incisors, showing significantly greater width (+0.29 mm; *p* = 0.005) and height (+0.32 mm; *p* = 0.011) compared with expert designs. AI-generated designs also yielded a substantially narrower buccal corridor than expert-generated designs (−3.64%; *p* < 0.001), representing a large effect size. No significant differences were identified in the width-to-height ratio or gingival zenith symmetry, and midline deviation was identical across all pairs. These findings indicate that AI-generated designs produced fuller anterior teeth and a narrower buccal corridor while maintaining comparable symmetry and midline parameters to expert-designed smiles.

### 3.8. Time Efficiency

AI-generated designs required substantially less time to complete compared with expert-generated designs. The mean time for AI designs was 30.82 ± 5.14 min, whereas expert-generated designs required 63.48 ± 14.12 min. Paired comparison demonstrated a highly significant reduction in time with the use of AI (t = −15.37, *p* < 0.001). Overall, AI reduced the time required for esthetic smile design by 51.46% relative to expert workflows, highlighting its efficiency advantage.

## 4. Discussion

To the authors’ knowledge, this study represents one of the first that has made a direct comparison of fully automated smile designs made by AI algorithms and designs made by a professional dentist using conventional digital smile design software. The results of this research have shown that even in a direct comparison, the designs made by artificial intelligence systems were well received on a variety of esthetic assessment parameters.

The findings strongly point to the rejection of the null hypothesis on the basis of significant differences that seem to favor the AI approach in many key parameters. Specifically, AI-generated designs attained higher scores on a validated esthetic index, received higher subjective ratings from both patients and experts, were strongly preferred by the patients, and were generated in less than half the time taken by the expert.

The main outcome variable was measured with the Dental Esthetic Screening Index (DESI), which showed that AI-designed cases were consistently higher than expert-designed cases. Meanwhile, significant advantages for AI were found in optimizing midline congruence, central incisor angulation, upper lip tooth display, gingival contour, and arch continuity, suggesting that the algorithm was able to operationalize predefined dentofacial esthetic parameters under standardized digital conditions [8,,18]. The only domain for which expert designs were found greater concerned the color harmony of teeth. This might be due to the subjective nature of clinical judgment behind tooth color matching, which is still not yet fully encapsulated by the AI automated processes. The generally lower DESI total score for AI designs confirms its ability to produce clinically excellent outcomes with adherence to established esthetic norms.

The subjective esthetic evaluations further cemented the advantage of AI-generated designs. Both patient and expert observers gave the AI outputs significantly higher VAS scores. The observed trend for patients to give higher absolute ratings than clinicians is consistent with reports in the literature, often explained by the fact that patients base their judgments on overall attractiveness, while experts focus on technical aspects and may notice flaws [19]. Importantly, the forced-choice preference test indicated a marked preference for AI designs among patients (69.7% vs. 30.3%), whereas the distribution in experts was close to equal. This difference highlights the possible mismatch between professional evaluation criteria and lay perceptions of what is esthetically pleasing, while this alignment may reflect the ability of AI systems to generate esthetic configurations consistent with contemporary digital design norms [20].

The objective geometric measurements then gave a concrete basis for these preferences. AI designs are invariably presented with central incisors of slightly increased width and height, yielding a statistically significant and substantially narrower buccal corridor. This particular feature has indeed been linked to increased smile attractiveness in previous studies [21,22]. The application of the above geometry by the AI in a consistent manner probably accounted for its high patient acceptance, while its maintained performance in the case of midline and gingival zenith symmetry confirmed its reliability in maintaining basic esthetic parameters.

From a clinical practice perspective, perhaps one of the most important results is the striking time efficiency. The AI system yielded a saving in design time of more than 51%, an enormous gain with absolutely no compromise on the quality, but rather an enhancement. This efficiency can streamline clinical workflows, improve productivity of practices, and make sophisticated digital smile design more accessible, which has been one of the major benefits identified in recent reviews of efficiency in digital dentistry [23].

The correlation analyses provide additional insight into the design processes: the strong positive correlation between AI design time and its DESI score suggests that more complex esthetic challenges require greater computational processing, mirroring the human experience. Second, the weak association of expert DESI scores with patient VAS ratings, compared to the stronger agreement for AI, would suggest that the AI “esthetic judgment” may more closely reflect patient-centric preferences than the technically oriented criteria used by expert clinicians.

The relatively lower DESI scores for the AI-designed categories of designs in the various esthetic fields indicate that the AI design process was able to apply the predefined criteria of midline, arch, and incisor positions to the designs. These factors and criteria form the basic components of smile design concepts and seem to be easily quantified or defined by the algorithmic process. The expert designs, however, seem to favor the DESI score for the factors of tooth color harmony, thus underlining the influence of clinical experience in the design process that goes beyond the geometrical and proportional principles.

A key finding was the striking difference in esthetic perception between the patients and the expert clinicians, best highlighted by the strong negative correlation (r = −0.92) between their VAS ratings for the expert-generated designs. This strong statistical relationship is indicative of a near-inverse relationship; the designs rated lower by experts were actually rated higher by patients, and vice versa. This quantifies a significant disconnection between professional criteria of evaluation and layperson perception.

Experts are trained to recognize minor deviations from idealized occlusal and proportional norms; as such, they may observe flaws that are either invisible or unimportant to the patients, who will more likely judge on the overall visual appeal and attractiveness of the smile. This divergence underlines a key problem in traditional esthetic dentistry: what the clinician considers a “gold-standard” outcome does not always equate with the patient’s “ideal.” Of particular note is that this disconnect was significantly reduced for AI-generated designs. This pattern may reflect alignment between AI-generated esthetic configurations and contemporary patient preferences.

Strengths of the study:

The main strengths of this study are its robust comparative design, with a predefined sample size that ensures adequate statistical power to detect clinically meaningful differences between groups. The paired nature of the analysis, in which each patient acted as their own control for both the AI and expert designs, removes inter-patient variability and thus allows for a direct comparison free of bias. The methodological rigor is high: this study used an extensive set of outcome measures, including a validated objective index (DESI), subjective ratings from two different stakeholder groups (expert prosthodontists and patients), objective digital measurements, and a time efficiency analysis. This multi-faceted approach provides a holistic evaluation of the digital smile design process. Thirdly, the blinding of the 20 expert raters to the origin of the designs and the randomization of design presentation mitigate assessment bias, strengthening the reliability of the subjective scores. The high inter-rater reliability (ICC > 0.9) further confirms the consistency of the expert evaluations. Finally, the inclusion of patient perspectives through both VAS ratings and a forced-choice preference task is a critical strength because it grounds the study’s conclusions in the ultimate endpoint of esthetic dentistry: patient satisfaction.

Limitations:

This study has several limitations that should be considered when interpreting the findings. First, the investigation was conducted in a virtual laboratory setting and evaluated proposed digital designs rather than clinically fabricated restorations. Therefore, the ability of AI-generated designs to translate into definitive restorations—considering occlusal dynamics, phonetics, material thickness, biomechanical principles, and biologic constraints—was not assessed.

Second, only a single AI platform (SmileFy) was evaluated; other systems may differ in algorithmic architecture and esthetic design philosophy, which may limit generalizability. In addition, AI outputs were analyzed in their fully automated form without expert refinement. In clinical practice, hybrid workflows incorporating clinician modification are likely and were not examined in this study.

Third, although the study was adequately powered, participants were recruited from a single center, which may restrict external validity.

Finally, a ceiling effect was observed in VAS ratings, with both patients and experts clustering near the upper limit of the scale. This likely reflects the idealized nature of digitally rendered designs, which lack clinical variables such as texture, surface characterization, and material imperfections. Such clustering may reduce the sensitivity of subjective scales to detect subtle differences between designs.

Clinical and Practical implications:

The efficacy of AI designs and their acceptance by patients point to various integration strategies. In a clinical setting, AI designs can act as an effective beginning point for smile design consultations, cutting down planning time while offering an esthetically pleasing base with patient approval. This option can prove particularly helpful in high clinics or with those who have limited expertise in esthetics.

A hybrid model of implementation, where AI designs are refined by experts, could provide the best possible outcome of efficiency and customization. Specialists will be left with the task of intricate details like characterization, occlusal support, and choice of material, and not basic tooth positions. This follows the newer concepts of digital dentistry, where standardization work will be done by computers, and experts will grapple with complexities.

The achievement of a 51.46% reduction in the time spent on design work is a major improvement in work efficiency. Practically applied, it may enable the clinician to work more on consultation with the patient and materials, and the consideration of the occlusal aspect, and less on the initial positioning of the teeth. A combination of the clinical and current digital models may be best achieved in a hybrid fashion regarding the positioning of the teeth and the support of the clinician, refashioning an initial design provided by the AI.

Future Prospects:

Future research should focus on validating the clinical translation of AI-generated smile designs by assessing their impact on restorative accuracy, material performance, and long-term patient satisfaction following definitive prosthodontic treatment. Comparative studies evaluating fully automated AI workflows, expert-only designs, and hybrid AI-assisted clinician refinement models are needed to determine the optimal integration strategy in clinical practice. Additionally, multicenter investigations involving diverse demographic and cultural populations would help clarify variability in esthetic preferences and improve the generalizability of algorithm-based design outputs. Further development of AI systems should incorporate advanced esthetic parameters, including surface texture, translucency, gingival morphology, and dynamic smile analysis, to enhance realism and clinical applicability. Longitudinal outcome studies integrating oral health-related quality-of-life measures are also warranted to establish the long-term functional and psychosocial benefits of AI-assisted digital smile design.

## 5. Conclusions

Within the limitations of this prospective paired virtual study, fully automated AI-generated smile designs demonstrated favorable esthetic performance, higher patient preference, and substantially improved time efficiency compared with expert-driven digital workflows. While expert-designed outputs maintained advantages in specific qualitative domains such as color harmony, AI systems consistently achieved comparable or superior results across multiple objective and subjective esthetic parameters. These findings indicate that AI-based smile design tools can serve as effective adjuncts to conventional digital workflows by enhancing efficiency and supporting patient-centered treatment planning. However, clinical validation studies incorporating definitive restorations, functional outcomes, and long-term follow-up are required before widespread clinical implementation can be recommended.

## Figures and Tables

**Table 1:** Comparative analysis of DESI item scores between AI-generated and expert-generated smile designs.

DESI Item		AI	Expert	Mean Difference	95% CI	*p*-Value	Cohen’s d
Extraoral Items	Congruence of facial and dental midline	1.18 ± 0.39	1.82 ± 0.77	−0.64	−1.00 to −0.27	0.001 *	−0.62
	CI angulation vs. facial midline	1.00 ± 0.00	1.61 ± 0.50	−0.61	−0.78 to −0.43	<0.001 *	−1.22
	Canine line vs. interpupillary line	1.58 ± 0.50	1.79 ± 0.42	−0.21	−0.49 to +0.06	0.13	−0.38
	Upper lip tooth display	1.15 ± 0.36	1.61 ± 0.50	−0.45	−0.73 to −0.16	0.003 *	−0.54
	Lower lip line vs. dental arch	1.03 ± 0.17	1.00 ± 0.00	+0.03	−0.01 to +0.08	0.11	+0.36
	Total	6.03 ± 0.77	8.82 ± 1.27	−2.24	−2.78 to −1.70	<0.001 *	−1.47
Intraoral Items	Gingival contour	1.03 ± 0.17	1.48 ± 0.51	−0.45	−0.73 to −0.16	0.003 *	−0.54
	Interdental papilla	1.58 ± 0.50	1.48 ± 0.51	+0.09	−0.09 to +0.27	0.32	+0.23
	Arch continuity	1.09 ± 0.29	1.73 ± 0.45	−0.64	−0.86 to −0.41	<0.001 *	−1.02
	Tooth angulation	1.27 ± 0.45	1.42 ± 0.50	−0.15	−0.35 to +0.05	0.14	−0.33
	Proximal contact	1.18 ± 0.39	1.42 ± 0.50	−0.24	−0.49 to +0.02	0.07 *	−0.46
	Tooth color harmony	1.39 ± 0.50	1.00 ± 0.00	+0.39	+0.22 to +0.57	<0.001 *	+0.79
	Width–height ratio	1.21 ± 0.42	1.39 ± 0.50	−0.18	−0.45 to +0.09	0.18	−0.24
	Total	8.75 ± 1.02	9.92 ± 1.11	−1.09	−1.60 to −0.59	<0.001 *	−0.77
Overall DESI Total		14.79 ± 1.63	18.73 ± 1.82	−3.33	−4.13 to −2.53	<0.001 *	−1.48

Lower DESI scores indicate better esthetic performance. Negative mean differences and negative effect sizes (Cohen’s d) reflect superior performance of the AI-generated designs compared with expert-generated designs. CI = confidence interval. * significance.

**Table 2:** VAS esthetic ratings for AI-generated and expert-generated designs.

Outcome	Experts (Mean ± SD)	Patients (Mean ± SD)	Mean Difference	95% CI	*p*-Value	Effect Size (Cohen’s d)
VAS for AI-Generated Designs	97.00 ± 0.66	97.59 ± 1.21	+0.79	0.51–1.06	<0.001	1.01
VAS for Expert-Generated Designs	96.21 ± 1.02	97.59 ± 1.21	+1.38	0.89–1.86	<0.001	0.80

Higher VAS scores indicate better perceived esthetics. Positive differences reflect higher ratings provided by patients compared with experts. Effect sizes ≥ 0.8 are considered large.

**Table 3:** Pearson correlation matrix among DESI, VAS ratings, buccal corridor width, and design time for AI- and expert-generated smile designs.

Measure	DESI-AI	DESI-EX	VAS-AI (Expert)	VAS-EX (Expert)	PtVAS-AI	PtVAS-EX	BC-AI	BC-EX	Time-AI	Time-EX
DESI-AI	1.00	0.81	0.66	0.17	0.02	0.92	0.75	−0.20	0.85	0.71
DESI-EX	0.81	1.00	0.53	0.24	−0.22	0.80	0.56	−0.45	0.54	0.55
VAS-AI (Expert)	0.66	0.53	1.00	0.17	0.00	−0.53	0.33	−0.33	0.20	0.10
VAS-EX (Expert)	0.17	0.24	0.17	1.00	−0.14	−0.92	−0.32	−0.11	−0.33	−0.31
Patient VAS—AI	0.02	−0.22	0.00	−0.14	1.00	0.26	−0.40	0.04	−0.09	−0.09
Patient VAS—EX	0.92	0.80	−0.53	−0.92	0.26	1.00	0.40	−0.41	0.83	0.66
Buccal Corridor—AI	0.75	0.56	0.33	−0.32	−0.40	0.40	1.00	−0.48	0.82	0.60
Buccal Corridor—EX	−0.20	−0.45	−0.33	−0.11	0.04	−0.41	−0.48	1.00	−0.63	−0.48
Time—AI	0.85	0.54	0.20	−0.33	−0.09	0.83	0.82	−0.63	1.00	0.78
Time—EX	0.71	0.55	0.10	−0.31	−0.09	0.66	0.60	−0.48	0.78	1.00

DESI = Dental Esthetic Screening Index; VAS = Visual Analog Scale; BC = Buccal Corridor; AI = artificial intelligence; EX = expert-generated design; Time = design generation time. Values represent Pearson correlation coefficients (r).

**Table 4:** Forced-choice preference between AI-generated and expert-generated designs.

Outcome	Experts	Patients	Mean Difference	*p*-Value
Preference for AI-Generated Designs	17/33 (51.5%)	23/33 (69.7%)	+18.2%	<0.001
Preference Expert-Generated Designs	16/33 (48.5%)	10/33 (30.3%)	−18.2%	<0.001

Forced-choice preference values represent the number and percentage of cases where AI or expert designs were favored. Chi-square test compares overall distributions of preferences.

**Table 5:** Objective esthetic measurements for AI-generated vs. expert-generated designs.

Objective Measurement	AI (Mean ± SD)	Expert (Mean ± SD)	Mean Difference (AI—Expert)	95% CI	*p*-Value	Effect Size (d)
Central Incisor Width (mm)	8.62 ± 0.42	8.33 ± 0.41	+0.29 mm	0.09 to 0.49	0.005 *	0.52
Central Incisor Height (mm)	10.12 ± 0.47	9.80 ± 0.45	+0.32 mm	0.08 to 0.56	0.011 *	0.47
W/H Ratio (%)	85.15 ± 2.11	85.02 ± 2.16	+0.13%	−0.55 to 0.80	0.706	0.07
Midline Deviation (mm)	0.00 ± 0.00	0.00 ± 0.00	0.00	—	—	—
Buccal Corridor (%)	13.21 ± 1.84	16.85 ± 2.03	−3.64%	−4.83 to −2.44	<0.001 *	−1.08
Gingival Zenith Symmetry (mm)	0.62 ± 0.38	0.83 ± 0.39	−0.21 mm	−0.47 to +0.05	0.109	−0.29

Mean difference represents AI minus expert measurements for each matched case. Positive values indicate higher measurements in AI-generated designs. Effect size (Cohen’s d) reflects the magnitude of paired differences, with 0.2, 0.5, and 0.8 typically interpreted as small, medium, and large effects, respectively. Dashes indicate measures with no variation between paired values. * Statistically significant at *p* < 0.05.

## Data Availability

The data presented in this study are available on reasonable request from the corresponding author.

## Abbreviations

The following abbreviations are used in this manuscript:

AI	Artificial Intelligence
CI	Confidence Interval
DESI	Dental Esthetic Screening Index
DSD	Digital Smile Design
ICC	Intraclass Correlation Coefficient
ML	Machine Learning
SD	Standard Deviation
STROBE	Strengthening the Reporting of Observational Studies in Epidemiology
VAS	Visual Analog Scale
3D	Three-Dimensional
